# Structure–function analyses of the bacterial zinc metalloprotease effector protein GtgA uncover key residues required for deactivating NF-κB

**DOI:** 10.1074/jbc.RA118.004255

**Published:** 2018-07-26

**Authors:** Elliott Jennings, Diego Esposito, Katrin Rittinger, Teresa L. M. Thurston

**Affiliations:** From the ‡ Section of Microbiology, MRC Centre for Molecular Bacteriology and Infection, Imperial College London, London SW7 2AZ and; the § Molecular Structure of Cell Signalling Laboratory, The Francis Crick Institute, 1 Midland Road, London NW1 1AT, United Kingdom

**Keywords:** Salmonella enterica, bacterial pathogenesis, metalloprotease, type III secretion system (T3SS), NF-κB, substrate specificity, bacterial effectors, GtgA, virulence factor

## Abstract

The closely related type III secretion system zinc metalloprotease effector proteins GtgA, GogA, and PipA are translocated into host cells during *Salmonella* infection. They then cleave nuclear factor κ-light-chain-enhancer of activated B cells (NF-κB) transcription factor subunits, dampening activation of the NF-κB signaling pathway and thereby suppressing host immune responses. We demonstrate here that GtgA, GogA, and PipA cleave a subset of NF-κB subunits, including p65, RelB, and cRel but not NF-κB1 and NF-κB2, whereas the functionally similar type III secretion system effector NleC of enteropathogenic and enterohemorrhagic *Escherichia coli* cleaved all five NF-κB subunits. Mutational analysis of NF-κB subunits revealed that a single nonconserved residue in NF-κB1 and NF-κB2 that corresponds to the P1′ residue Arg-41 in p65 prevents cleavage of these subunits by GtgA, GogA, and PipA, explaining the observed substrate specificity of these enzymes. Crystal structures of GtgA in its apo-form and in complex with the p65 N-terminal domain explained the importance of the P1′ residue. Furthermore, the pattern of interactions suggested that GtgA recognizes NF-κB subunits by mimicking the shape and negative charge of the DNA phosphate backbone. Moreover, structure-based mutational analysis of GtgA uncovered amino acids that are required for the interaction of GtgA with p65, as well as those that are required for full activity of GtgA in suppressing NF-κB activation. This study therefore provides detailed and critical insight into the mechanism of substrate recognition by this family of proteins important for bacterial virulence.

## Introduction

The virulence of many pathogenic Gram-negative bacteria relies partially on the functions of type III secretion systems (T3SS).^3^ These syringe-like apparatuses assemble across both bacterial and host cell membranes and translocate bacterial proteins termed “effectors” into host cells. To date, six catalytically-active zinc metalloprotease T3SS effector proteins have been identified. These include the highly related family of effectors GtgA, GogA, and PipA (Fig. S1) from pathogenic *Salmonella enterica* serovars (1), NleC and NleD from enteropathogenic and enterohemorrhagic *Escherichia coli* (EPEC and EHEC) (2), as well as RipAX2 from the plant pathogen *Ralstonia solanacearum* (3). All six effectors contain the short metal-binding motif “HE*XX*H,” which contains two histidine residues that coordinate the active-site zinc, as well as a catalytically important glutamate residue. This glutamate activates a zinc-bound water molecule for nucleophilic attack on the carbonyl group of the substrate peptide (4). Whereas NleD cleaves and inactivates the mitogen-activated protein kinase signaling proteins JNK and p38 to inhibit activator protein-1 (AP-1)–dependent gene transcription (2), GtgA, GogA, PipA, and NleC cleave NF-κB subunits, thereby inhibiting activation of NF-κB–dependent innate immune signaling (1, 2, 5–7). The substrate of RipAX2 is unknown; however, the catalytically important glutamate residue in the HE*XX*H motif is required for the induction of a hypersensitive response in the nonhost wild eggplant *Solanum torvum* (3). HopH1 from *Pseudomonas syringae* (8) and XopG from *Xanthomonas arboicole* (9), which share 46 and 48% amino acid sequence identity with RipAX2, also contain a HE*XX*H motif; however, HopH1 and XopG have not yet been shown to be catalytically active.

The NF-κB signaling pathway regulates the expression of a large number of genes involved in inflammation, immunity, cell proliferation, and survival (10). The pathway can be activated by pattern recognition receptors, and following a cascade of post-translational modifications, it ultimately leads to the nuclear import of NF-κB transcription factors. Subsequent binding of NF-κB transcription factors to specific palindromic nucleotide sequences in the promoter and enhancer regions of target genes, collectively termed “κB-sites,” can lead to both transcriptional activation and repression (10). NF-κB transcription factors consist of homo- and heterodimers of the NF-κB subunits p65, RelB, cRel, NF-κB1 (p105/p50), and NF-κB2 (p100/p52). All of these contain a Rel homology region (RHR) toward their N terminus that is responsible for DNA binding and dimerization. p65, RelB, and cRel also contain a transcriptional activation domain near their C termini, whereas p105 and p100 contain glycine-rich regions and multiple copies of ankyrin repeats that are removed during processing to form the p50 and p52 subunits. Structural analyses of NF-κB RHR dimers in complex with DNA have revealed that the RHR contains two immunoglobulin-like domains, the N-terminal domain (NTD) and the C-terminal dimerization domain (DD), separated by a short flexible linker (11–13). In each dimer subunit, a flexible loop in the NTD, as well as a limited number of residues in the flexible linker and dimerization domain, contacts DNA bases in the major groove of one-half of the palindromic recognition sequence. Electrostatic interactions between positively charged residues in the NTD and DD and the negatively charged DNA phosphate backbone also occur (14).

GtgA, GogA, PipA, and NleC all cleave the NTD of p65 despite the fact that the sequence identity shared between members of the GtgA family and NleC is low (<20%). NleC primarily cleaves p65 between residues Cys-38 and Glu-39 (2), whereas GtgA, GogA, and PipA cleave between residues Gly-40 and Arg-41 (1). NleC has also been reported to cleave p65 between residues Pro-10 and Ala-11 (5), although the cleavage efficiency relative to the Cys-38/Glu-39 cleavage site is reduced, and the significance is unclear (15). GtgA, GogA, and PipA also cleave RelB but not p105/p50 or p100/p52 (1), and NleC cleaves cRel (6), RelB (2, 16), and p50 (6, 7, 16). The molecular basis for the different substrate specificities of NleC and GtgA, GogA, and PipA is not clear. It is also unknown whether NleC can cleave p100/p52 and whether GtgA, GogA, and PipA can cleave cRel.

GtgA, GogA, PipA, and NleC do not share significant sequence identity with other known zinc metalloproteases. However, the structure of NleC demonstrates that the catalytic core retains the structural topology of other members of the Zincin superfamily (16, 17) (Fig. S2); the active-site cleft, at the bottom of which sits the HE*XX*H-containing active-site helix, is bifurcated by an N-terminal subdomain (NSD) and a C-terminal subdomain (CSD), and the three C-terminal strands of a β-sheet in the NSD form a ψ-loop motif. In this motif, two external antiparallel β-strands are zippered together by an internal β-strand that runs parallel to the N-terminal β-strand in the motif (Fig. S2).

Although the crystal structure of NleC has been solved, the mechanism of substrate recognition by which GtgA, GogA, PipA, and NleC recognize and cleave the NTD of NF-κB subunits is not fully understood. Interestingly, NleC's active–site cleft is similar in shape to the DNA major groove and is highly negatively charged, leading to the hypothesis that NleC's substrate specificity is determined by mimicking the shape and charge of DNA (16, 17).

In this study, we confirm that GtgA, GogA, and PipA cleave p65 and RelB but not NF-κB1 (p105/p50) and NF-κB2 (p100/p52) and identify cRel as an additional substrate of these proteases. Furthermore, we demonstrate that NleC cleaves all five NF-κB subunits, reporting for the first time that NleC cleaves the NF-κB2 (p100/p52) subunit. Mutational analysis of residues in close proximity to the peptide bond in p65 cleaved by GtgA, GogA, and PipA (Gly-40/Arg-41) revealed that these zinc metalloprotease effectors show strong P1′ site selectivity. Residues in p105/p50 and p100/p52 that correspond to the P1′ residue Arg-41 in p65 are not conserved, explaining the substrate specificity of these proteases. We also present the crystal structure of Zn^2+^-bound GtgA and Zn^2+^-free GtgA in complex with the NTD of p65. Similar to NleC, the active-site cleft of GtgA is highly negatively charged, and the active-site cleft mimics the shape of the DNA major groove. Accordingly, the complex structure reveals that GtgA interacts with residues in p65 that are required for the interaction of p65 with DNA. Furthermore, mutational analysis of GtgA residues that interact with the p65 NTD in the complex structure identified residues important for GtgA to interact with p65, as well as for its proteolytic activity. This study therefore provides novel insight into the mechanism of substrate recognition for GtgA, GogA, and PipA.

## Results

### Substrate specificity of GtgA, GogA, PipA, and NleC

**Figure 1.**
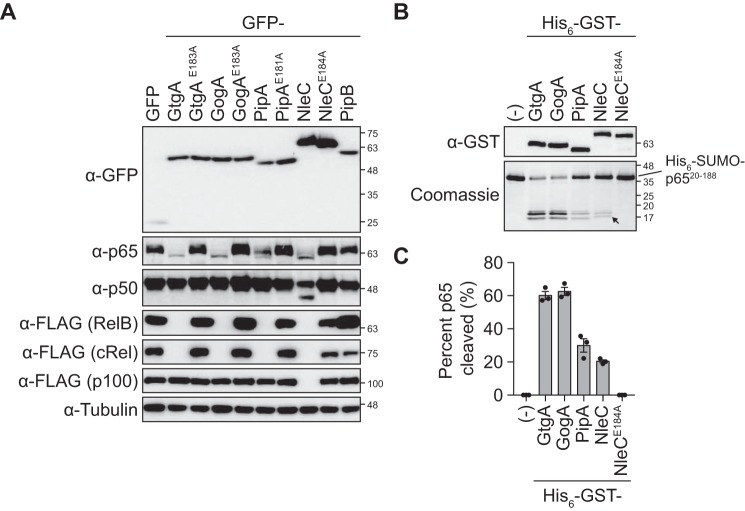
**Substrate specificity of GtgA, GogA, PipA, and NleC.** *A,* immunoblot analysis of 293ET cells cotransfected with plasmids encoding the indicated GFP-tagged effector proteins and FLAG-tagged NF-κB subunits. The abundance of endogenous p65 and p50 was analyzed using anti-p65 and anti-p50 antibodies, whereas FLAG-tagged RelB, cRel, and p100 were detected using an anti-FLAG antibody. An anti-tubulin antibody was used as a loading control. Immunoblots shown are representative of three independent experiments. *B,* 5 μm His_6_–SUMO–p65(20–188) was incubated with 0.1 μm of the indicated His_6_–GST-tagged effector protein for 5 h at 37 °C. The reaction was quenched by the addition of 2× Laemmli buffer, and proteins were separated and visualized by SDS-PAGE followed by Coomassie Blue staining. The *arrow* indicates the larger cleavage product generated by NleC relative to GtgA, GogA, and PipA. Immunoblot analysis using an anti-GST antibody was done to confirm equal amounts of each GST-tagged effector protein. The Coomassie Blue-stained polyacrylamide gel and immunoblot are representative of three independent experiments. *C,* quantification of His_6_–SUMO–p65(20–188) cleavage in *B*. Data are presented as percent cleavage relative to control sample (−) and represent the mean ± S.E. of three independent experiments, for which individual data points are indicated.

It has been reported previously that GtgA, GogA, and PipA cleave the NF-κB subunits p65 and RelB but not p105/p50 or p100/p52 (1), which is in contrast to NleC, which cleaves p65, p50, cRel, and RelB (2, 5–7). However, a systematic analysis of the NF-κB subunits cleaved by each effector has never been reported. We therefore transfected 293ET cells with plasmids encoding GFP-tagged GtgA, GogA, PipA, NleC, or the unrelated *Salmonella* T3SS effector PipB (18) as a negative control, and we determined the cleavage of endogenous p65 and p50 and ectopically expressed FLAG epitope–tagged RelB, cRel, and p100 by Western blotting. Endogenous p65 was cleaved in 293ET cells expressing GtgA, GogA, PipA, and NleC, whereas endogenous p50 was only cleaved in cells expressing NleC (Fig. 1*A* and Fig. S3). Additionally, FLAG-tagged RelB and cRel were undetectable in GtgA-, GogA-, PipA-, and NleC-expressing cells, despite being detected in GFP- and PipB-expressing cells, identifying cRel as an additional substrate of GtgA, GogA, and PipA. Finally, FLAG-tagged p100 was not detected in NleC-expressing cells, whereas the abundance of FLAG–p100 in cells expressing GtgA, GogA, and PipA remained indistinguishable from control conditions. Therefore, p100/p52 represents an additional substrate of NleC.

These data demonstrate that whereas the EPEC/EHEC T3SS effector NleC cleaves all five NF-κB subunits, the *Salmonella* T3SS effectors GtgA, GogA, and PipA show clear substrate specificity toward p65, RelB, and cRel. Mutation of the glutamate residue in the metal-binding HE*XX*H motif of each effector to alanine, abrogated NF-κB subunit cleavage, confirming that the catalytic activity of these effectors was required for function (Fig. 1*A* and Fig. S3).

### p65 N-terminal domain is directly cleaved by GtgA, GogA, and PipA

Truncation analysis of p65 has shown that the NTD of p65 (residues 19–187) is the smallest structural region of p65 cleaved by NleC (19). To determine whether the NTD of p65 is also directly cleaved by GtgA, GogA, and PipA, we purified residues 20–188 of p65 fused with an N-terminal His_6_–SUMO expression tag, and following incubation with GST-tagged effector proteins, we analyzed cleavage by SDS-PAGE and Coomassie Blue staining. Similar to NleC, His_6_–SUMO–p65(20–188) was directly cleaved by GtgA, GogA, and PipA, with the greatest amount of cleavage product detected following incubation with GtgA (60% cleavage) and GogA (61%) relative to PipA (32%) and NleC (21%) (Fig. 1, *B* and *C*). The site in p65 cleaved by GtgA, GogA, and PipA (Gly-40/Arg-41) (1) is two amino acids downstream of the primary cleavage site targeted by NleC (Cys-38/Glu-39) (2). Our results are consistent with this, as the fragment that migrated the furthest through the polyacrylamide gel (indicated by the *arrow* in Fig. 1*B*) was larger when His_6_–SUMO–p65(20–188) was cleaved by NleC. Comparison of the cleavage products produced by cleavage of His_6_–SUMO–p65(20–188) and His_6_–SUMO–p65(20–291) by GtgA, demonstrated that this band is the C-terminal fragment (Fig. S4).

### Molecular basis for GtgA, GogA, and PipA substrate specificity

Schechter and Berger nomenclature (20) provides a system for describing the interactions of a peptide substrate with the active site of a protease. The substrate residues are designated by their position relative to the scissile bond; N-terminal residues are referred to as P1, P2, P3, and P4, whereas those on the C-terminal side are referred to as P1′, P2′, P3′, and P4′. The “subsites” in the active-site cleft that interact with the substrate residues are named in a similar manner (S1, S2, and S3 and S1′, S2′, and S3′, etc).

**Figure 2.**
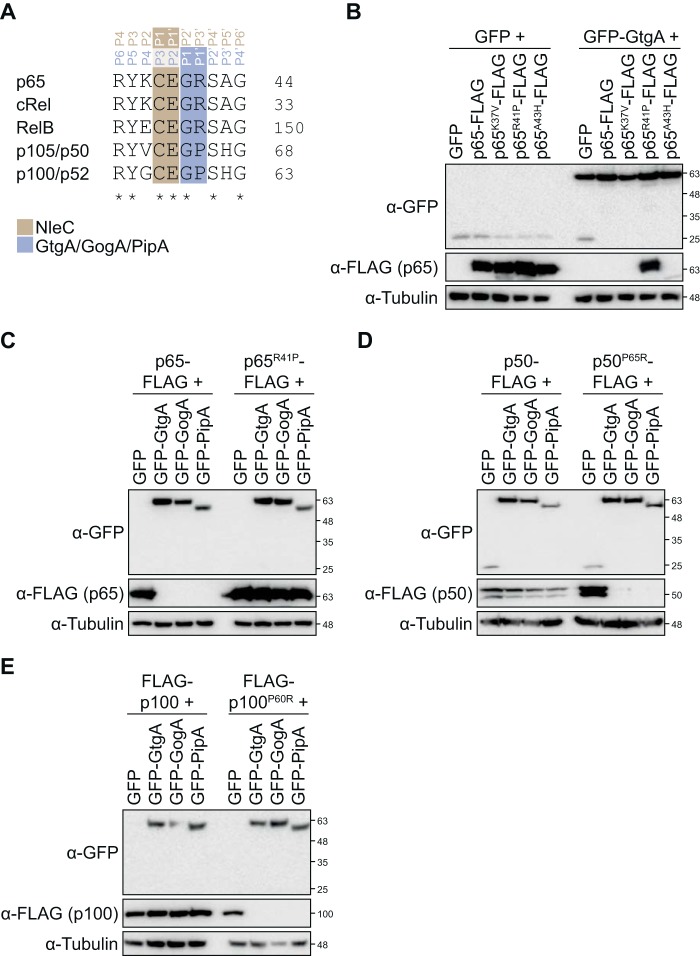
**P1′ site is a critical determinant of GtgA, GogA, and PipA substrate specificity.** *A,* sequence alignment of residues in close proximity to the GtgA, GogA, PipA, and NleC cleavage site in p65 and the corresponding residues in cRel, RelB, p105/p50, and p100/p52. Uniprot numbers are Q04206, Q04864, Q01201, P19838, and Q00653. The cleavage sites targeted by each enzyme is highlighted. *B–E,* immunoblot analysis of 293ET cells transiently transfected with plasmids encoding the indicated GFP-tagged effector and FLAG-tagged NF-κB subunit. Immunoblotting of whole-cell lysates was done with an anti-GFP and anti-FLAG antibody. Lysates were also blotted with an anti-tubulin antibody as a loading control. Immunoblots are representative of at least three independent experiments.

Sequence alignment of the amino acids surrounding the peptide bond cleaved by GtgA, GogA, and PipA shows that three residues in p105/p50 (Val-61, Pro-65, and His-67) and p100/p52 (Gly-56, Pro-60, and His-62) that correspond to the P4, P1′, and P3′ sites in p65 are not conserved (Fig. 2*A*). As these two NF-κB subunits are not cleaved by the GtgA family of effectors, we hypothesized that these residues are important for substrate recognition. To test this hypothesis, we mutated each nonconserved residue in p65 to the corresponding residue in p50. FLAG-tagged p65 variants and GFP–GtgA were expressed ectopically in 293ET cells, and whole-cell lysates were immunoblotted with an anti-FLAG antibody. WT FLAG-tagged p65 and the p65 variants K37V and A43H were not detected in cells expressing GtgA despite equal expression in GFP-expressing cells (Fig. 2*B*) suggesting that these variants were efficiently cleaved by GtgA. However, the abundance of p65^R41P^–FLAG was indistinguishable between cells expressing GFP alone or GFP–GtgA. Similarly, the abundance of p65^R41P^–FLAG was unchanged in GogA- and PipA-expressing cells relative to GFP-expressing cells (Fig. 2*C*). The inverse mutation in p50 and p100 had the opposite effect; there was no difference in the abundance of WT FLAG-tagged p50 or p100 in GFP-, GtgA-, GogA-, or PipA–expressing cells, but p50^P65R^ and p100^P60R^ were no longer detected in cells expressing GtgA, GogA, or PipA, despite being detected in cells expressing GFP alone (Fig. 2, *D* and *E*). These data demonstrate that the P1′ site (residue Arg-41 in p65) is a critical determinant of substrate specificity for GtgA, GogA, and PipA and that these enzymes are unable to cleave p105/p50 and p100/p52 because of a proline present at the corresponding site in these NF-κB subunits. In contrast, NleC is able to cleave all of the NF-κB subunits because the cleavage site it targets, including the critical P1′ site (16, 17), is conserved (Fig. 2*A*).

### Structure of the T3SS effector GtgA

To gain further insight into the mechanism of substrate recognition by GtgA, we solved the crystal structure of GtgA alone and in complex with the p65 NTD (residues 20–188). To prevent cleavage of p65 by GtgA during crystallization, GtgA was inactivated by mutating the glutamate residue in the HE*XX*H motif to a glutamine (E183Q). We also removed the first 19 N-terminal residues of GtgA because the N-terminal translocation signal of T3SS effectors, usually consisting of the first 15–25 amino acids, is frequently unstructured and can prevent crystallization (21, 22). This had no apparent effect on the enzymatic activity of GtgA *in vitro* (Fig. S5).

**Figure 3.**
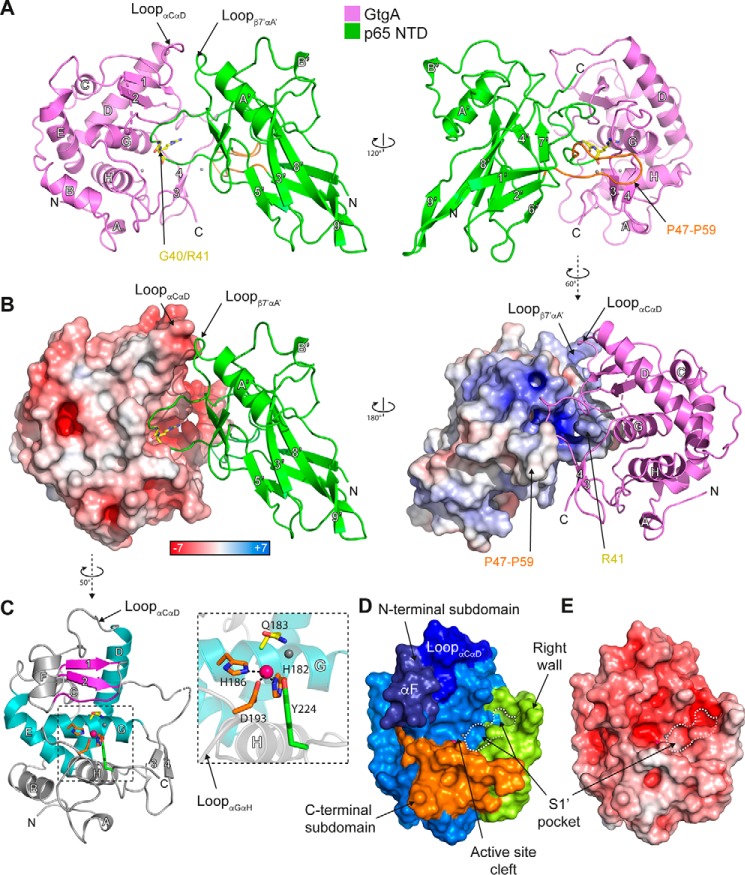
**Crystal structures of GtgA alone and in complex with the p65 N-terminal domain.** *A,* crystal structure of Zn^2+^-free GtgA(20–228)^E183Q^ in complex with p65(20–188). The α-helices and β-strands in GtgA are labeled A to H and 1 to 4, respectively. In the p65 NTD, the α-helices are labeled A′ and B′, and the β-strands are labeled 1′ to 9′. The p65 cleavage site residues Gly-40/Arg-41 are colored *yellow* and shown in a *stick* representation. p65 residues Pro-47 to Pro-59 are colored *orange*. The chloride ions are shown as *gray spheres. B,* surface and cartoon representation of GtgA in complex with the p65 NTD. GtgA (*left*) and p65 (*right*) are colored according to the electrostatic surface potential (positive *blue*, negative *red*), as calculated using Adaptive Poisson-Boltzmann Solver (APBS) in PyMOL (44). *C,* crystal structure of Zn^2+^-bound GtgA(20–228)^E183Q^. The α-helices and β-strands in GtgA are labeled A to H and 1 to 4, respectively. The Zincin-like catalytic core is colored as in Ref. 4; α-helices are colored *teal*, and the β1β2 β-sheet and the active-site upper rim residues are colored *purple*. The zinc ion and chloride ion are shown as *pink* and *gray spheres*, respectively. A close-up view of the catalytic zinc ion and active-site residues in a *stick* representation are shown as an *inset*. Zinc-coordinating residues are colored *orange*, Tyr-224 *green,* and Gln-183 *yellow. D,* surface representation of GtgA(20–228)^E183Q^ colored according to the active-site subdomains. With the exception of the right wall, which is colored in *green*, the N-terminal subdomain is colored in different shades of *blue*, and the C-terminal subdomain is shown in *orange. E,* solvent-accessible surface representation colored according to the electrostatic surface potential (positive *blue*, negative *red*) of the structure of GtgA(20–228)^E183Q^.

The crystal structure of Zn^2+^-free GtgA(20–228)^E183Q^ in complex with the p65 NTD was solved at 2.1 Å resolution by molecular replacement using the structure of p65 NTD (PDB 2RAM) as search template (Fig. 3, *A* and *B*; Table 1). GtgA(20–228)^E183Q^–p65(20–188) crystals belong to space group *P*2_1_2_1_2_1_ with a single heterodimer in the asymmetric unit (a.u.). Residues 20–27 and 137–145 and the C-terminal residue Asn-228 are not visible in the electron density of GtgA(20–228)^E183Q^ nor are the two C-terminal residues of p65(20–188) (residues Arg-187 and Ala-188).

**Table 1:** **Crystallographic data collection and refinement statistics** The highest-resolution shell values are given in parentheses.

	GtgA(20–228)^E183Q^	GtgA(20–228^)E183Q^–p65(20–188)
**PDB ID**	6GGO	6GGR

**Data collection statistics**		
Wavelength (Å)	0.9686	0.9159
Resolution range (Å)	70.28–2.6	55.93–2.097
Highest-resolution range (Å)	2.72–2.6	2.172–2.097
Space group	*I*121	*P*2_1_2_1_2_1_
Cell dimensions		
*a, b, c* (Å)	95.78 40.68 112.161	39.39 85.83 111.87
α, β, γ (°)	90 94.0027 90	90 90 90
Total reflections	43,450 (4336)	714,811 (61672)
Unique reflections	13,578 (1345)	22,948 (2217)
Multiplicity	3.2 (3.2)	31.1 (27.8)
Completeness (%)	99.8 (99.8)	99.0 (97.7)
Mean *I*/σ (*I*)	5.3 (1.3)	13.9 (3.4)
Wilson *B*-factor	59.91	32.36
*R*-merge	0.102 (0.617)	0.1894 (1.419)
*R*-meas	0.122 (0.743)	0.1927 (1.446)
*R*-pim	0.067 (0.408)	0.03482 (0.2735)
*CC*1/2	0.992 (0.919)	0.995 (0.946)

**Refinement statistics**		
Reflections used in refinement	13,394 (1304)	22,770 (2208)
Reflections used for *R*-free	594 (63)	1129 (107)
*R*-work	0.2175 (0.4119)	0.2061 (0.2901)
*R*-free	0.2564 (0.4379)	0.2520 (0.3280)
No. of non-hydrogen atoms	3243	3028
Macromolecules	3182	2877
Ligands	4	2
Solvent	57	149
Protein residues	397	359
Root mean square (bonds) (Å)	0.003	0.006
Root mean square (angles) (°)	0.51	0.65
Ramachandran favored (%)	98.71	98.87
Ramachandran allowed (%)	1.29	1.13
Ramachandran outliers (%)	0	0
Rotamer outliers (%)	0.56	0.31
Clashscore	2.44	2.49
Average *B*-factor	93.53	49.83
Macromolecules	93.95	50.2
Ligands	72.71	30.71
Solvent	71.91	43.02

The structure of Zn^2+^-bound GtgA(20–228)^E183Q^ was solved at 2.6 Å resolution by molecular replacement using the coordinates of GtgA in complex with p65 (Fig. 3, *A* and *B*; Table 1). No electron density was visible for residues 20–24, 157, and 158 and the C-terminal Asn-228. GtgA(20–228)^E183Q^ crystals belong to space group *I*121 with two molecules in the a.u. that overlap with a root mean square deviation (RMSD; Cα of residues 27–154 and 160–227) of 0.2 Å. The two molecules are covalently linked by a disulfide bond between Cys-44 of each chain. This is likely a crystallization artifact, as the elution volume of GtgA(20–228)^E183Q^ following size-exclusion chromatography was indicative of a monomeric protein (Fig. S6), and the disulfide bond is absent in the structure of the GtgA–p65 complex.

GtgA, in both its apo-form (Fig. 3*C*) and in complex with p65 (Fig. 3, *A* and *B*), has a globular structure, with the catalytic core assuming a Zincin-like fold (4). The active-site helix (αG) sits at the bottom of a cleft formed between an upper NSD and a lower CSD (Fig. 3*D*). The NSD includes two “backing helices” (αD and αE) that fold into a V-shape, as well as the active-site helix and a two-stranded β-sheet formed by strands β1 and β2. The CSD, which forms the lower rim of the active-site cleft, includes all residues following the active-site helix, including the αH-helix. An extra segment, between residues Asn-152 and Asp-175, frames the right-hand side of the active-site cleft and will be referred to herein as the right wall (Fig. 3*D*).

In the GtgA apo structure, the catalytic zinc ion is tetrahedrally coordinated by the Nϵ2 atoms of each histidine in the canonical zinc-metalloprotease motif ^182^HE*XX*H^186^, Asp-193 in the loop connecting α-helices G and H, and a chloride ion (see “Experimental procedures” and Fig. 3*C*). As observed in the crystal structure of other Zincins such as Asticin (PDB 1AST) (23), it is likely that in WT GtgA, the chloride ion would be replaced with a catalytically important water molecule. In the structure of NleC published previously (PDB 4Q3J) (16), the catalytic zinc ion is additionally coordinated by a tyrosine residue (Tyr-227). The corresponding residue in GtgA, Tyr-224, is in close proximity (3.9 Å) to the zinc ion (Fig. 3*B*), but it is too distant to be a coordinating residue. NleC variants with mutations in the corresponding zinc-ligating residues are catalytically inactive, except the tyrosine mutant (Y227A), which, although active, had reduced activity toward p65 *in vitro* (17).

**Figure 4.**
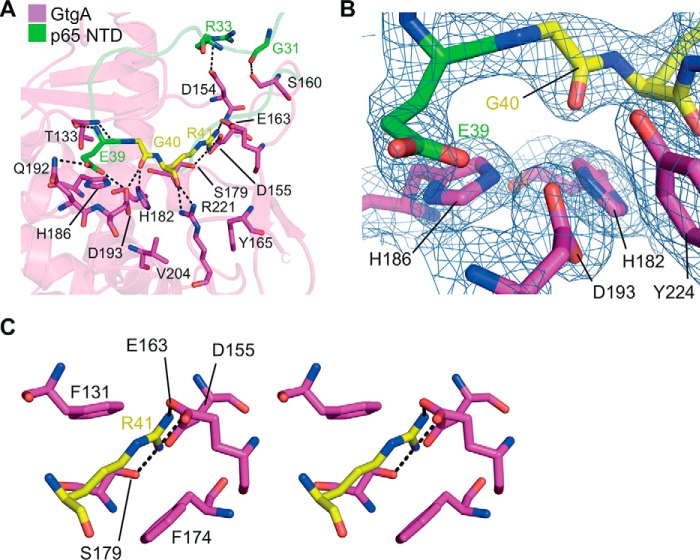
**GtgA and p65 N-terminal domain interaction.** *A,* close-up view of the interactions between the GtgA active-site cleft and the p65 NTD. GtgA is colored *purple*, and p65 is colored *green* with the exception of the p65 cleavage site residues (Gly-40/Arg-41), which are colored *yellow. B,* 2*F_o_* − *F_c_* electron density map contoured at 1σ of the GtgA zinc-coordinating residues (His-182, His-186, and Asp-193) and Tyr-224, as well as p65 residues Glu-39 and Gly-40. *C,* wall-eye stereo view of the p65 P1′ residue Arg-41 (colored *yellow*) inserted into the GtgA S1′ pocket. *A* and *C, black dashed lines* represent hydrogen bonds.

In other Zincins, the NSD β-sheet normally contains three to five strands (4), with the three C-terminal β-strands forming a ψ-loop motif. The substrate and the lowest β-strand of the ψ-loop (β-strand 2 in Fig. S2), which forms the upper rim of the active-site cleft, run antiparallel, with their interaction stabilized by backbone hydrogen bonds similar to those seen in a β-sheet. In GtgA, the ψ-loop motif is incomplete as, in both the complex and apo structures, the lowermost strand residues Phe-131 to Val-135, which form the upper rim, are not in the β-sheet (Fig. 3*C*). However, hydrogen bonds are still formed between this strand and p65 in the complex structure (Fig. 4), suggesting a mechanism of substrate recognition similar to other Zincins (4).

The active site of GtgA is surrounded by a large negatively charged surface (Fig. 3*E*) formed by αF, the upper rim residues, and the loop connecting helices αC and αD, which folds down over the incomplete ψ-loop (Fig. 3*B*). The electrostatic surface potential of GtgA might therefore mimic the negative charge of the DNA–phosphate backbone as a mechanism of substrate recognition.

### Structure of GtgA in complex with the p65 N-terminal domain

GtgA interacts with the p65 NTD via a large interface with a buried surface area of 1081 Å^2^. Most of the residues in p65 that interact with GtgA, including the p65 cleavage site, are in the loop connecting strands β1′ and β2′, with secondary binding interactions represented by the positively charged surface of the loop connecting strand β7′ and helix αA′ in p65 and the negatively charged surface of the loop connecting helices αC and αD in GtgA (Fig. 3*B*). The αF helical segment present in the GtgA apo structure is invisible in the electron density of the GtgA–p65 complex. An overlap of the apo-GtgA with the GtgA chain in the complex structures demonstrates that this region clashes sterically with residues in the p65 NTD chain (Fig. S7).

Within the active-site cleft of GtgA, the backbone of p65 Glu-39 is hydrogen-bonded to the backbone of Thr-133 of GtgA, whereas its side chain forms hydrogen bonds with the side chains of GtgA residues Gln-192 and Asp-193 (Fig. 4*A*). Despite the absence of the active-site zinc atom in the complex structure of GtgA–p65 (Fig. 4*B*), the carbonyl oxygen of the P1 residue Gly-40 is positioned above the zinc-coordinating site, hydrogen-bonded with GtgA His-182 and Asp-193 (Fig. 4*A*). This arrangement is similar to that seen in the structure of a catalytically inactive, Zn^2+^-bound form of the zinc metalloprotease insulin-degrading enzyme in complex with a peptide substrate (PDB 2G54) (24) and the structure of Asticin in complex with a transition state analog (PDB 1QJI) (25).

The P1′ residue Arg-41 inserts into the negatively charged S1′ pocket (Fig. 3, *B*, *D,* and *E*) formed by the N-terminal portion of the upper rim and the right wall of the active-site cleft. The side chain of p65 Arg-41 is sandwiched by hydrophobic contacts in between the aromatic rings of Phe-131 and Phe-174, with its guanidino group directly hydrogen-bonded to the side chains of GtgA Asp-155, Glu-163, and Ser-179 (Fig. 4, *C* and *D*). The backbone carbonyl oxygen of p65 Arg-41 is hydrogen-bonded to the guanidino atoms of GtgA Arg-221. Above the S1′ pocket, Gly-31 and Arg-33 in p65 hydrogen-bond with residues Ser-160 and Asp-154, respectively, on the right wall of the GtgA active-site cleft (Fig. 4*A*). In addition, p65 residues Pro-47 to Pro-59 in the loop connecting strands β1′ and β2′ form an extended horseshoe shape that folds around the back of the GtgA right wall and is stabilized by inter-chain long-range van der Waals contacts (Fig. 3*A*).

The structure of the p65 NTD in complex with a catalytically-inactive form of GtgA reported here (Fig. 3*A*) is similar to the existing structures of the p65 NTD in complex with various DNA sequences with Cα RMSD values between 0.92 (PDB 5U01.B) (26) and 1.43 Å (PDB 1RAM.A) (11). However, compared with the majority of p65–DNA complex structures, the side chains of the P2 residue Glu-39 and the P1′ residue Arg-41 are flipped ∼180° so that Glu-39 is hydrogen-bonded to Gln-192 of GtgA in the loop connecting helices αG and αH, and the Arg-41 guanidino group extends into the S1′ pocket (Fig. 4).

### Mutagenic analysis of the GtgA–p65 NTD interaction

To assess the roles of the observed interactions between GtgA and the p65 NTD, we generated catalytically inactive GtgA variants in which residues that hydrogen-bond to residues in the p65 NTD via their side chains were mutated to alanines (D154A, D155A, D159A, S160A, E163A, S179A, Q192A, and R221A). The ability of these variants to interact with p65 was then determined in a luminescence-based mammalian interactome mapping (LUMIER) binding assay. In this assay, p65 fused via its C terminus to *Renilla* luciferase and was expressed ectopically in 293ET cells. These cells were subsequently lysed and incubated with the indicated purified GST-tagged GtgA^E183A^ variant or GST alone as a negative control, and a GST-pulldown using GSH Sepharose beads was performed. Fold binding of the GtgA^E183A^ variants to p65–*Renilla* was then determined by measuring the amount of luminescence after GST-pulldown relative to the input using a *Renilla* luciferase assay.

**Figure 5.**
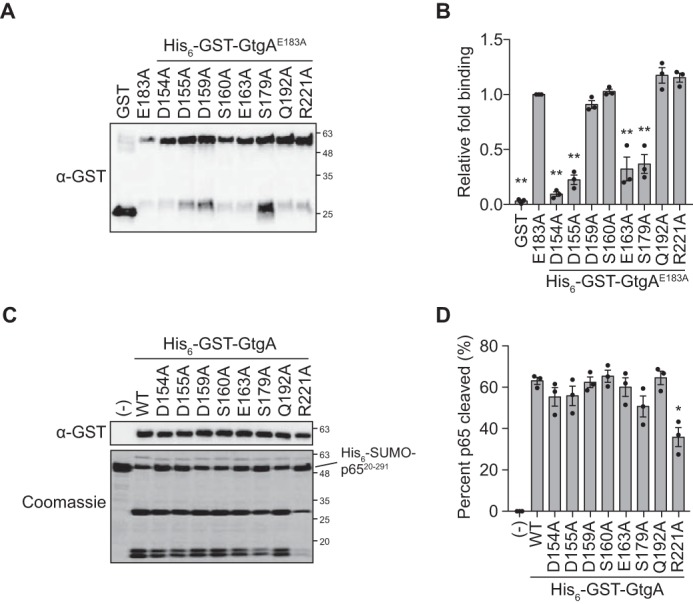
**Mutational analysis of GtgA–p65 interacting residues.** *A* and *B,* LUMIER-binding assay. His_6_–GST-tagged GtgA variants were incubated with the post-nuclear supernatant of 293ET cells expressing p65 fused via its C terminus to the *Renilla* luciferase. Following elution, immunoblot analysis was performed using an anti-GST antibody (*A*), and *Renilla* luciferase activity was measured to calculate the relative fold binding (*B*). Immunoblot is representative of three independent experiments. Data are presented as the fold change in *Renilla* luciferase activity relative to His_6_–GST–GtgA^E183A^ and represents the mean ± S.E. of three independent experiments, for which individual data points are indicated. Statistical significance was computed between GtgA^E183A^ and each GtgA^E183A^ variant (*, *p* < 0.05; **, *p* < 0.01, ordinary one-way ANOVA with post hoc Dunnett's multiple comparisons test). *C,* 5 μm His_6_–SUMO–p65(20–291) was incubated with 0.1 μm of the indicated His_6_–GST–GtgA variant for 5 h at 37 °C. The reaction was then quenched by the addition of 2× Laemmli buffer, and proteins were separated and visualized by SDS-PAGE followed by Coomassie Blue staining. Immunoblot analysis using an anti-GST antibody was done to confirm equal amounts of each GST-tagged effector protein. The Coomassie Blue-stained polyacrylamide gel and immunoblots are representative of three independent experiments. *D,* quantification of His_6_–SUMO–p65(20–291) cleavage in *C*. Data are presented as percentage cleavage relative to control sample and represent the mean ± S.E. of three independent experiments, for which individual data points are indicated. Statistical significances were computed between WT and each GtgA variant (*, *p* < 0.05; **, *p* < 0.01, ordinary one-way ANOVA with post hoc Dunnett's multiple comparisons test).

p65–*Renilla* was pulled down with GST–GtgA^E183A^ but not GST alone, demonstrating that catalytically inactive GtgA forms a complex with p65 (Fig. 5, *A* and *B*). The interaction of GtgA^E183A^ variants D159A, S160A, Q192A, and R221A was indistinguishable from GtgA^E183A^ showing that individual mutation of these residues does not affect substrate recognition. In contrast, the interaction of p65–*Renilla* with GtgA^E183A^ variants D154A, D155A, E163A, and S179A was significantly diminished relative to GtgA^E183A^ (Fig. 5, *A* and *B*). In the GtgA–p65 complex structure, Asp-155, Glu-163, and Ser-179 hydrogen-bond to the guanadino group of p65 P1′ residue Arg-41, whereas GtgA residue Asp-154 hydrogen-bonds to p65 residue Arg-33 (Fig. 4*A*). These data are therefore consistent with our previous observations that GtgA shows strong P1′ site selectivity (Fig. 2) and additionally identifies that GtgA residue Asp-154 is required for the formation of a stable GtgA–p65 complex.

To examine whether GtgA mutants with a reduced ability to form a stable complex with p65 result in a negative impact on the proteolytic activity of GtgA, we generated the same GtgA variants with an intact HE*XX*H zinc metalloprotease motif and analyzed the ability of these variants to cleave His_6_–SUMO–p65(20–291) in an *in vitro* cleavage assay. His_6_–SUMO–p65(20–291) was incubated at a 50:1 molar ratio with the GtgA variants for 5 h at 37 °C, and cleavage efficiency analyzed by SDS-PAGE and Coomassie Blue staining (Fig. 5*C*). Quantification in Fig. 5*D* shows that ∼60% of His_6_–SUMO–p65(20–291) was cleaved by WT GtgA. The cleavage of His_6_–SUMO–p65(20–291) by six out of the eight GtgA variants analyzed (Fig. 5, *A* and *B*) was indistinguishable from WT GtgA. However, the catalytic activity of GtgA^R221A^ was significantly reduced relative to WT GtgA (Fig. 5, *C* and *D*) despite GtgA^E183A/R221A^ showing no reduction in its interaction to p65 (Fig. 5*B*). There was also a slight reduction in the catalytic activity of GtgA^S179A^; however, relative to WT GtgA, this was nonsignificant.

**Figure 6.**
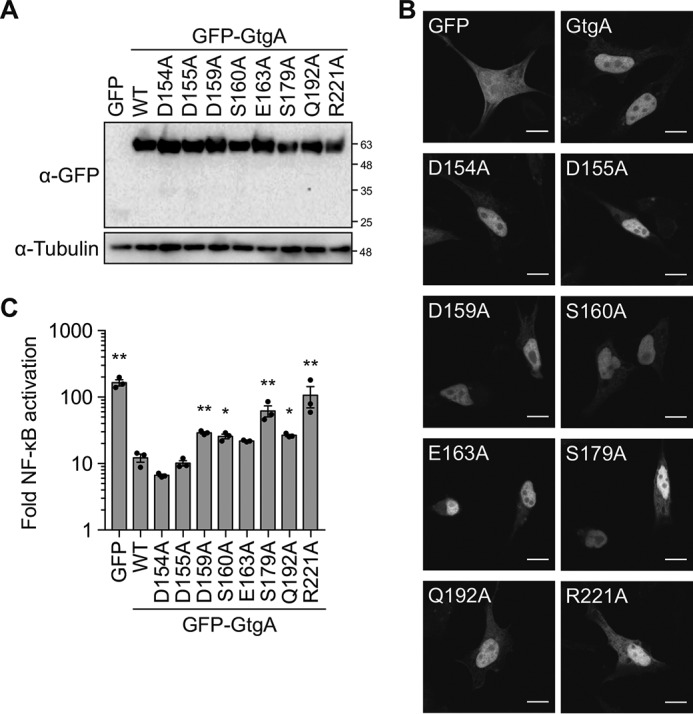
**NF-κB inhibition by GtgA variants.** *A,* immunoblot analysis of 293ET cells co-transfected with plasmids encoding an NF-κB–dependent firefly luciferase, a constitutively expressed *Renilla* luciferase, and GFP or the indicated GFP–GtgA variant. 293ET cells were stimulated with 20 ng/ml TNFα for 8 h. *B,* confocal microscopy images of HeLa cells transiently transfected with plasmids encoding GFP or the indicated GFP–GtgA variant. *Scale bar,* 10 μm. *C,* luciferase activity was measured in cell lysates from the experiment shown in *A*. Data are presented as the fold change in NF-κB reporter activity between unstimulated and TNFα-stimulated 293ET cells and represent the mean ± S.E. of three independent experiments, for which individual data points are indicated. Statistical significances were calculated between WT and each GtgA variant (*, *p* < 0.05; **, *p* < 0.01, ordinary one-way ANOVA with post hoc Dunnett's multiple comparisons test).

Next, we tested the ability of GtgA variants to inhibit TNFα-induced activation of an NF-κB–dependent luciferase reporter in 293ET cells. All of the mutants analyzed were expressed to a similar level as WT GtgA as determined by Western blot analysis of whole-cell lysates (Fig. 6*A*), and the introduced mutations had no effect on the nuclear localization of GtgA (Fig. 6*B*).

As expected, TNFα-induced NF-κB reporter activation was strongly inhibited in 293ET cells expressing WT GtgA relative to GFP-expressing cells (Fig. 6*C*). Consistent with the results of the *in vitro* cleavage assay (Fig. 5, *C* and *D*), the ability of GtgA^R221A^ to inhibit activation of the NF-κB luciferase reporter was substantially reduced (Fig. 6*C*). Notably, GtgA^S179A^ also had a reduced capability to inhibit NF-κB reporter activity, which could have arisen from its diminished interaction to p65 (Fig. 5*B*) and reduced catalytic activity *in vitro* (Fig. 5, *C* and *D*). Finally, there was a small but significant increase in NF-κB reporter activation in cells expressing GtgA point mutants D159A, S160A, or Q192A relative to cells expressing WT GtgA (Fig. 6*C*). Therefore, these findings identify residues within GtgA that are required for the suppression of host NF-κB activity. Together, these results provide molecular insight into the substrate recognition and activity of this family of T3SS effectors.

## Discussion

Here, we report that the zinc metalloprotease T3SS effector proteins GtgA, GogA, and PipA from *S. enterica* cleave a subset of NF-κB subunits comprising p65, RelB, and cRel, whereas NleC from EPEC/EHEC cleaves all five NF-κB subunits. Although cleavage of some NF-κB subunits by these effectors has been shown previously, this is the first report to systematically analyze the cleavage specificity of each protease on all five NF-κB subunits. Furthermore, mutagenic analysis of residues in close proximity to the p65 peptide bond cleaved by GtgA, GogA, and PipA, revealed that the P1′ site in p65 (residue Arg-41) is a key determinant of substrate specificity. A proline residue is present at the corresponding site in both NF-κB1 and NF-κB2, explaining why these NF-κB subunits are not cleaved by the GtgA family. In contrast, the P1′ site in p65 cleaved by NleC (Glu-39) is conserved across all five NF-κB subunits. In addition, we present the crystal structures of GtgA alone and in complex with the N-terminal domain of p65. From this, we identify residues within the active-site cleft of GtgA that are required for a stable interaction with p65. Whereas the majority of these mutants retain the ability to inhibit the NF-κB reporter, suggesting that secondary interactions are sufficient for GtgA to cleave its substrate, we also identified GtgA mutants that displayed reduced function while retaining WT levels of binding to p65. Overall, our study provides further molecular insight into the mechanism of substrate recognition by these enzymes.

A DALI search for structures homologous to GtgA identified NleC as GtgA's closest structural homolog (PDB 4Q3J; *Z* score = 10.7, RMSD = 3.0). GtgA was also identified to share weak structural homology to the zinc metalloprotease domains of tetanus toxin (PDB 5N0C; *Z* score = 7.1, RMSD = 3.4) and botulinum neurotoxin type E (PDB 3FFZ; *Z* score = 7.0, RMSD = 3.3). Superimposition of GtgA and NleC revealed that the Zincin-like catalytic cores of the two enzymes are similar suggesting that the catalytic mechanism of GtgA and NleC is the same. For monometallic proteases, it is generally accepted that following substrate binding, the carbonyl group of the substrate scissile bond binds to the catalytic zinc ion. A zinc-bound water molecule then performs a nucleophilic attack on the carbonyl carbon atom to form a gem-diolate tetrahedral intermediate that is stabilized by either a proximal histidine, arginine, or tyrosine residue (4). The glutamate in the short metal-binding motif HE*XX*H is essential for this nucleophilic attack as it activates the zinc-bound water molecule. In the GtgA apo structure, Tyr-224 is 3.9 Å from the active-site zinc, suggesting that this residue, which is conserved in GogA, PipA, and NleC, stabilizes the tetrahedral intermediate. The corresponding residue in NleC (Tyr-227) is required for optimal NleC catalytic activity *in vitro* (17).

**Figure 7.**
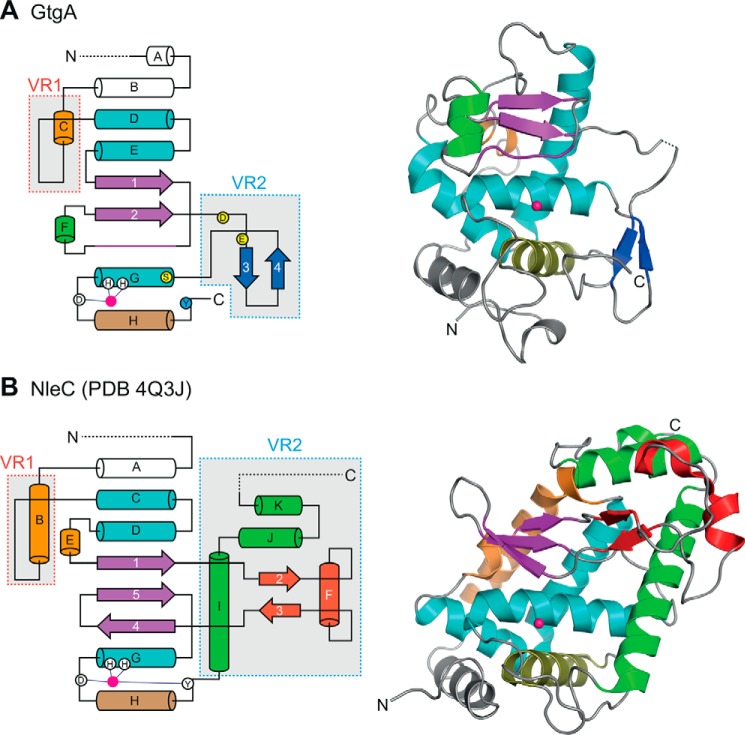
**Structural comparison of GtgA and NleC.** Topological and cartoon representations of GtgA (*A*) and NleC (*B)* (PDB 4Q3J). The Zincin-like catalytic core is colored as in Fig. 3*C*; α-helices are colored *teal*, and the β1β2 β-sheet and the active-site upper rim residues are colored *purple*. Variable features are colored in *orange, blue, red,* and *green,* and the catalytic zinc ions are shown as *pink spheres. Dashed lines* represent either disordered residues or regions outside the crystallized constructs. In the topological diagrams, zinc-coordinating residues are shown as *white circles,* and the active-site zinc is shown as a *pink circle*. GtgA residue Tyr-224 is shown as a *blue circle*, and GtgA S1′ pocket residues Asp-155, Glu-163, and Ser-179 are shown as *yellow circles*.

Although the catalytic cores of GtgA and NleC are similar, structural differences are apparent in two separate regions: variable region 1 and variable region 2 (VR1 and VR2) (Fig. 7). In GtgA, VR1 contains the loop connecting helices αC and αD, the negatively charged surface of which forms the uppermost section of the active-site cleft and interacts with complementarily charged residues in p65. The corresponding region in NleC does not form part of the active-site cleft and is therefore unlikely to be important for NF-κB subunit recognition by NleC (Fig. 7*B*). VR2 in GtgA is represented by the right wall of the GtgA active-site cleft (Fig. 3*C* and 7*A*) and includes the S1′ pocket residues Asp-155 and Glu-163, which are important for GtgA–p65 stable complex formation. NleC VR2 also includes the right wall of the NleC active site, which is formed by helix αI (residues 229–251) (Fig. 7*B*). NleC residue Arg-239 in helix αI points toward the catalytic zinc ion and occupies the same structural position as the GtgA S1′ pocket. The presence of a positively charged side chain in this position might partially explain why the P1′ residue recognized by NleC is Glu-39, whereas the P1′ residue recognized by GtgA, GogA, and PipA is Arg-41. Intriguingly, residue Arg-239 in NleC helix αI is conserved in the NleC homolog AIP56, whereas most of the other residues in helix αI are not. AIP56 is a secreted AB toxin of *Photobacterium damselae piscicida,* the catalytic domain of which functions identically to NleC by cleaving p65 between residues Cys-38 and Glu-39 (27).

The active-site cleft of NleC is hypothesized to mimic the DNA major groove in shape and is also highly negatively charged, with residues Glu-115, Glu-150, and Glu-153 required for the efficient cleavage of p65 by NleC (16, 17). The active-site cleft of GtgA is similarly negatively charged, implying that the mechanism of substrate recognition by GtgA and NleC is similar. However, the surface distribution of glutamate and aspartate residues in GtgA differs from that in NleC, suggesting that the angle by which the p65 NTD interacts with the active-site cleft of each enzyme is slightly tilted. This leads to the positioning of a different peptide bond above the catalytic zinc ion and thus directly affects substrate specificity.

**Figure 8.**
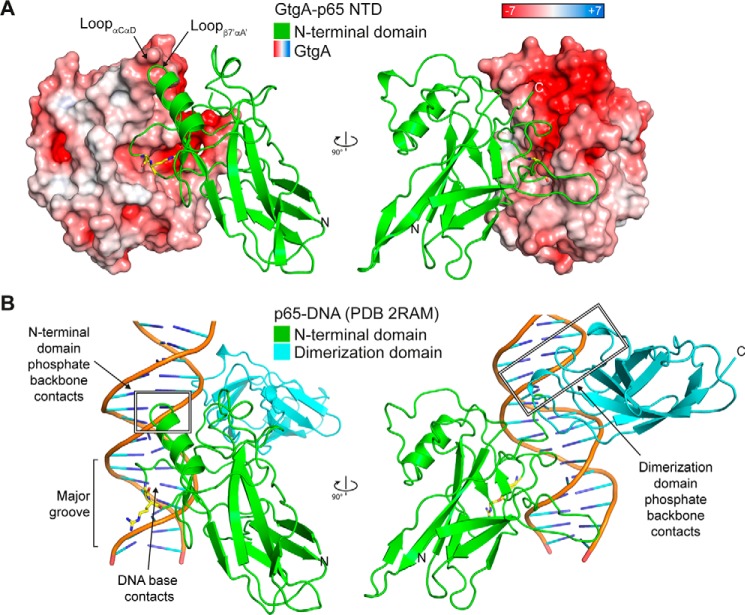
**DNA mimicry by GtgA.** *A,* surface representation of GtgA colored according to its electrostatic surface potential (positive *blue*, negative *red*), in complex with the NTD of p65. *B,* cartoon representation of the p65 RHR in complex with DNA (PDB 2RAM) (11). In both panels, the p65 cleavage site residues (Gly-40/Arg-41) are represented as *yellow sticks*.

Positively charged p65 residues Lys-122, Lys-123, and Arg-124, which interact with the negatively charged phosphate backbone of DNA in published p65–DNA complex structures (Fig. 8*B*) (11, 14), interact in the GtgA–p65 NTD complex structure, with the negatively charged surface of the loop connecting αC and αD in GtgA (Figs. 3*A* and 8*A*). Published p65–DNA complex structures also show that positively charged residues in the flexible linker and dimerization domain interact with the negatively charged DNA–phosphate backbone (Fig. 8*B*) (11, 14). In the GtgA–p65 NTD complex structure, the C terminus of the p65 NTD points toward a second negatively charged groove on the surface of GtgA that is adjacent to the active-site cleft (Fig. 8*A*). Furthermore, superimposition of a single p65 RHR (residues 19–291) in complex with DNA (PDB 2RAM.A), with the p65 NTD (residues 20–188) in complex with GtgA, places residues in the p65 flexible linker and dimerization domain that interact with the DNA–phosphate backbone, in close proximity to this second negatively-charged groove. Although the p65 dimerization domain is not required for cleavage of p65 by GtgA *in vitro* (Fig. 1*B*), it is conceivable that GtgA utilizes a bi-modal recognition mechanism involving electrostatic interactions between two negatively charged grooves on the surface of GtgA and the two immunoglobulin-like domains in the RHR of NF-κB subunits.

Mutagenic analysis revealed that in addition to the zinc-coordinating residues, GtgA residue Arg-221 is important for efficient GtgA catalytic activity *in vitro* and for the inhibition of NF-κB signaling in 293ET cells despite not being required for stable complex formation. The side chain of Arg-221 is sandwiched between two hydrophobic residues, Val-204 in helix αH and the aromatic ring of Tyr-165 in strand β3 (Fig. 4), such that it probably stabilizes the right wall of the active-site cleft as well as the C-terminal tail of GtgA. Mutation of Arg-221 to an alanine could therefore have perturbed the structural integrity of these regions and affected the positioning of Tyr-224 relative to the active-site zinc.

In addition to Arg-221, residues Asp-159, Ser-160, Ser-179, and Gln-192 were important for complete GtgA-mediated inhibition of NF-κB activity in 293ET cells (Fig. 6). With the exception of Asp-159, all of these residues are conserved in PipA; therefore, this mutagenic analysis does not explain why PipA is less efficient than both GtgA and GogA at cleaving p65 *in vitro* (Fig. 1, *B* and *C*). However, residues that form helix αF in GtgA are not conserved in PipA (Fig. S1), and homology models of GogA and PipA generated using the GtgA crystal structure show that the surface of helix αF in PipA, which forms the left portion of the N-terminal subdomain, is not negatively charged (Fig. S8).

During *Salmonella* infection, cleavage of the NF-κB subunits p65, RelB, and cRel by GtgA, GogA, and PipA inactivates these NF-κB subunits thereby inhibiting NF-κB–dependent gene transcription (1). In addition to a reduction in full-length p65 protein, the N-terminal fragment of p65 (p65(1–38)), produced after cleavage by NleC, interacts with and prevents the nuclear import of the transcriptional coactivator ribosomal protein S3 (RPS3) thereby further inhibiting a specific subset of NF-κB–dependent genes whose expression requires RPS3 (15). As cleavage of p65 by the GtgA family produces a very similar product (p65(1–40)), we predict that RPS3-dependent gene transcription will be inhibited during *Salmonella* infection.

Despite this, it is probable that the differing substrate specificities of the GtgA family, compared with NleC, result in the modulation of different subsets of NF-κB–dependent genes in *Salmonella-*infected and EPEC/EHEC-infected cells, respectively. A number of NF-κB–responsive genes have been shown to be regulated by specific NF-κB homo- and heterodimers (28–30). For example, the p50 homodimer represses interferon-stimulated response elements (31), and the p52 homodimer, in complex with Bcl3, activates expression of genes, including *IL-10*, *MIP-1*α, and *MIP-1*β (30).

In summary, we have defined the molecular and structural basis of substrate specificity for the zinc metalloprotease T3SS effector proteins GtgA, GogA, PipA, and NleC. The crystal structure of GtgA in complex with the N-terminal domain of the NF-κB subunit p65 provides further evidence that NF-κB–degrading zinc metalloprotease T3SS effector proteins recognize the RHR of NF-κB subunits by mimicking the shape and negative charge of the DNA–phosphate backbone.

## Experimental procedures

### Plasmids

The ORFs encoding *Salmonella* Typhimurium strain ATCC 14028s and *E. coli* strain O127:H6 E2348/69 effector proteins were amplified from their respective genomic DNA and cloned into pCMV (a modified version of pEGFP-N1 (Clontech)) as GFP fusion proteins using PciI and NotI. The ORFs of *gtgA, gogA, pipA*, and *nleC* (Uniprot: A0A0F6AZI6, A0A0F6B537, A0A0F6AZQ0, and B7UNX4) were then subcloned into the pETM30 vector (32) with a tobacco etch virus (TEV) protease–cleavable N-terminal hexahistidine and GST tag. pETM30–GtgA(20–228) was generated in a similar manner.

pcDNA3.RelB–cFLAG and pcDNA3.FLAG–Rel were obtained from Addgene (plasmid numbers 20017 and 27253). The ORFs of *RELA* and *NFKB2* (Uniprot: Q04206 and Q00653) amplified from 293ET cDNA were ligated into pcDNA3.cFLAG digested with HindIII and BamHI and pCMV.FLAG digested with PciI and NotI, respectively. To generate pcDNA3.p65–*Renilla*, the FLAG epitope tag between the BamHI and EcoRI restriction sites in pcDNA.cFLAG was excised and replaced with the *Renilla* ORF amplified from the plasmid pRL-TK. The ORF of *RELA* was then ligated into pCDNA.cRenilla digested with HindIII and BamHI. pE-SUMO.p65(20–188) and pE-SUMO.p65(20–291) were generated by amplifying the ORF of murine *RelA* (Uniprot: Q04207) from RAW264.7 cDNA and ligating into pE-SUMO (LifeSensors) digested with BsaI and NotI. All point mutants were generated using overlap extension PCR.

### Protein purification

*E. coli* BL21 PC2 cells (33) transformed with a plasmid encoding the desired protein were grown in LB broth at 37 °C to an *A*_600_ of 0.6 supplemented with 50 μg/ml kanamycin. For the expression of zinc metalloproteases, LB broth was additionally supplemented with 100 μm ZnCl_2_. Protein expression was then induced with 0.5 mm isopropyl β-d-1-thiogalactopyranoside for 16 h at 18 °C, and cells were harvested by centrifugation. Pellets were suspended in lysis buffer (50 mm HEPES, pH 7.5, 300 mm NaCl, 2 μg/ml DNase I, 10 μg/ml lysozyme, 2 mm MgCl_2_, 0.5 mm tris(2-carboxyethyl)phosphine (TCEP), 10% (v/v) glycerol, 1 mm phenylmethylsulfonyl fluoride) and lysed by sonication using a Bandelin Sonoplus sonicator. Next, the bacterial lysate was clarified by centrifugation at 38,000 × *g* for 1 h at 4 °C. The supernatant was then passed through a gravity flow column containing either GSH–Sepharose 4B resin (GE Healthcare) or HisPur^TM^ Ni-NTA resin (ThermoFisher Scientific). GSH–Sepharose resin was washed with buffer A (50 mm Tris, pH 8, 300 mm NaCl), and Ni-NTA resin was washed with buffer A supplemented with 20 mm imidazole, pH 8.0.

For biochemical assays, proteins were then eluted from the resin using buffer A supplemented with either 25 mm reduced GSH for GSH–Sepharose or 500 mm imidazole, pH 8.0, for Ni-NTA. Proteins were dialyzed overnight at 4 °C into 25 mm HEPES, pH 7.5, 150 mm NaCl, and 0.5 mm TCEP before concentration to ∼5 mg/ml using Amicon Ultra-15 (MWCO 10 kDa) centrifugation filters (Merck). Aliquots were frozen in liquid N_2_ and stored at −80 °C for later use.

For crystallization, proteins were eluted from the resin following overnight incubation with 0.5 mm TCEP and TEV protease or the SUMO protease ULP1. TEV cleavage of the His_6_–GST tag left the amino acids GAM prior to the first residue of GtgA, whereas ULP1 cleavage of the His_6_–SUMO tag left a single alanine prior to the first residue of p65. Finally, the proteins were purified on a HiLoad 16/60 Superdex 75 size-exclusion column (GE Healthcare) in 25 mm HEPES, pH 7.5, 150 mm NaCl, and 0.5 mm TCEP. Peak fractions of the appropriate purity were pooled and concentrated to between 10 and 25 mg/ml using Amicon Ultra-15 (MWCO 10 kDa) centrifugation filters (Merck). Aliquots were frozen in liquid nitrogen and stored at −80 °C for later use. Protein concentrations were determined by UV absorption at 280 nm using theoretical absorbance coefficients calculated using ProtParam (34).

### Crystallization and structure determination

Proteins purified by affinity- and size-exclusion chromatography were thawed and used in crystallization trials conducted using the sitting-drop vapor diffusion method. A Mosquito liquid handling robot (TTP Labtech) was used to dispense 100 nl of protein solution and 100 nl of reservoir solution into each drop. The crystallization trays were then incubated at 20 °C, and crystal growth was monitored using a Rock Imager 1000 (Formulatrix). The GtgA(20–228)^E183Q^–p65(20–188) complex, prepared by mixing 175 μm of each protein immediately prior to crystallization, was crystallized in a buffer containing 0.1 m Tris, pH 8.3, 0.5 m LiCl, and 32.5% (w/v) PEG 6000. Plate-like crystals appeared within 48 h. GtgA(20–228)^E183Q^, at a concentration of 24 mg/ml, crystallized, forming needle crystals within 12 h in a buffer containing 0.1 m Tris, pH 8.5, 25% (v/v) isopropyl alcohol, and 20% (w/v) PEG 3350. Crystals were flash-frozen in liquid N_2_ without cryoprotectant prior to data collection. Data sets were collected at Diamond Light Source (Oxford, UK) at beamlines i24 and i04. Data for the GtgA(20–228)^E183Q^–p65(20–188) complex were processed with DIALS (http://dials.diamond.ac.uk/).^4^ Apo GtgA(20–228)^E183Q^ data were integrated with DIALS (http://dials.diamond.ac.uk/)^4^ and scaled and merged with Aimless (35).

Initial phases of GtgA(20–228)^E183Q^ in complex with p65(20–188) were calculated by molecular replacement using the structure of p65(19–188) (PDB 2RAM) (11) as a search model in PHASER (36). Repeated cycles of manual chain building in Coot (37), automated chain building using PhenixAutoBuild wizard (38), and refinement with Phenix (39) and REFMAC5 (40) were then used to model the structure of GtgA(20–228)^E183Q^ in complex with p65(20–188). The final structural model was assessed and validated using the Phenix-integrated MolProbity tool (41). A model of GtgA(20-228)^E183Q^ was generated in a similar manner with the exception that the initial phases were calculated by molecular replacement using the structure of GtgA(20–228)^E183Q^ from the GtgA–p65 NTD complex. The electron density characteristics surrounding the catalytic zinc in GtgA(20–228)^E183Q^ suggested the presence of a heavier coordinating element other than a water molecule. Because it was the most abundant anion in the crystallization conditions, we placed a chloride ion in the metal coordination shell, eliminating the large residual peak in the difference map resulting from placing a coordinating solvent molecule. The statistics for the X-ray data collection and structural refinement are summarized in Table 1. The interface between GtgA(20–228)^E183Q^ and p65(20–188) was analyzed using PDBsum (42). SWISS-MODEL was used to generate structural homology models of GogA and PipA (43). All structural figures were prepared in PyMOL (Schrödinger, LLC).

### Cell culture and DNA transfections

293ET cells (gift from Felix Randow) and HeLa cells were maintained in Dulbecco's modified Eagle's medium (Sigma) supplemented with 10% fetal calf serum (Sigma) at 37 °C in 5% CO_2_. Cells were transfected using Lipofectamine 2000 (Life Technologies, Inc.) as per the manufacturer's instructions.

### SDS-PAGE and immunoblotting

1.5 × 10^5^ 293ET cells seeded into 24-well plates were transfected with 200 ng of pCMV.GFP-(effector) and 200 ng of a plasmid expressing the indicated FLAG-tagged NF-κB subunit. 24 h post-transfection, cells were washed with PBS and then lysed by the addition of 2× Laemmli buffer to each well. Whole-cell lysates were separated by SDS-PAGE using polyacrylamide gels of varying percentages (8–14%) and transferred onto PVDF membranes. Immunoblotting was done using mouse anti-FLAG (M2, Sigma), rabbit anti-GFP (G10362, Invitrogen), goat anti-p65 (sc-372-G, Santa Cruz Biotechnology), mouse anti-p50 (4D1, Biolegend), rabbit anti-GST (G7781, Sigma), and mouse anti-tubulin (E7, DHSB). HRP-conjugated anti-rabbit (Dako), anti-mouse (Dako), and anti-goat (Sigma) secondary antibodies were used for detection on a Chemidoc^TM^ Touch Imaging System (Bio-Rad).

### In vitro cleavage assays

5 μm of purified His_6_–SUMO–p65 were mixed with 0.1 μm His_6_–GST–effector fusion proteins in 40 μl of reaction buffer (25 mm HEPES, pH 7.5, 150 mm NaCl, 0.5 mm TCEP). Following a 5-h incubation at 37 °C, the reaction was quenched by the addition of 40 μl of 2× Laemmli buffer. Substrate cleavage was analyzed by SDS-PAGE followed by staining with PageBlue protein staining solution (ThermoFisher Scientific).

### LUMIER binding assay

1.5 × 10^5^ 293ET cells seeded in 24-well plates were transfected with 300 ng of pcDNA.p65-cRenilla. 24 h post-transfection, cells were washed with PBS and lysed in LUMIER lysis buffer (20 mm Tris, pH 7.4, 150 mm NaCl, 5% (v/v) glycerol, 0.1% (v/v) Triton X-100, and cOmplete EDTA-free protease inhibitor mixture (Roche Applied Science)). Post-nuclear supernatants were isolated by centrifugation at 16,000 × *g* for 10 min at 4 °C and then incubated with 5 μg of purified GST or His_6_–GST–GtgA variants and 5 μl of GSH–Sepharose 4B resin (GE Healthcare) for 2 h at 4 °C. The resin was subsequently washed four times with LUMIER lysis buffer, and GST was eluted using 10 mm GSH in *Renilla* luciferase assay lysis buffer (Promega). *Renilla* luciferase activity was then measured using the *Renilla* luciferase assay system (Promega) and a Tecan Infinite 200 PRO plate reader. The *Renilla* activity of each sample was measured in duplicate.

### NF-κB luciferase reporter assays

1.5 × 10^5^ 293ET cells were seeded into 24-well plates, 24 h before transfection with 100 ng of NF-κB–responsive luciferase reporter plasmid (pPRDII:luc), 40 ng of *Renilla* luciferase expression plasmid (pRL-TK), and 150 ng of pCMV.GFP–GtgA variants. 24 h after transfection, cells were stimulated with 20 ng/ml human TNFα (Sigma) for 8 h. Luciferase activity in cell lysates was then measured using the Dual-Luciferase reporter assay system (Promega) and a Tecan Infinite 200 PRO plate reader. The activity of the firefly luciferase was normalized to the activity of the *Renilla* luciferase, and then the fold change relative to unstimulated cells was calculated for each GtgA variant.

### Confocal microscopy

2 × 10^5^ HeLa cells, seeded onto glass coverslips 24 h before use, were transfected with 250 ng of each pCMV.GFP–GtgA variant. 24 h post-transfection, cells were washed once with PBS and then fixed with 3% paraformaldehyde (PFA) for 15 min at room temperature. Fixed cells were then washed a further three times with PBS, PFA autofluorescence quenched with 50 mm NH_4_Cl, and coverslips then mounted onto glass sides using Aqua-Poly/mount (Polysciences, Inc.). Coverslips were imaged using an LSM 710 inverted confocal microscope (Zeiss GmbH).

### Statistical analysis

Statistical differences between multiple groups was calculated using a one-way analysis of variance (ANOVA) and post hoc Dunnett's test (*, *p* < 0.05; **, *p* < 0.01) in GraphPad Prism version 7.

## Author contributions

E. J. and T. L. T. conceptualization; E. J. and D. E. investigation; E. J. writing-original draft; E. J., D. E., K. R., and T. L. T. writing-review and editing; D. E., K. R., and T. L. T. supervision; D. E. methodology; K. R. resources.

## Supplementary Material

Supporting Information

## Footnotes

4 Please note that the JBC is not responsible for the long-term archiving and maintenance of this site or any other third party hosted site.

## Footnotes

3 The abbreviations used are:

T3SS: type III secretion system
RMSD: root mean square deviation
EPEC: enteropathogenic *E. coli*
EHEC: enterohemorrhagic *E. coli*
TCEP: tris(2-carboxyethyl)phosphine
Ni-NTA: nickel-nitrilotriacetic acid
NTD: N-terminal domain
NSD: N-terminal subdomain
CSD: C-terminal subdomain
DD: dimerization domain
RHR: Rel homology region
PDB: Protein Data Bank
LUMIER: luminescence-based mammalian interactome mapping
GST: glutathione *S*-transferase
ANOVA: analysis of variance
a.u.: asymmetric unit
TEV: tobacco etch virus
PFA: paraformaldehyde.
