# Supplementary material for: Structure–function analyses of the bacterial zinc metalloprotease effector protein GtgA uncover key residues required for deactivating NF-κB
Source: J Biol Chem. 2018 Jul 26;293(39):15316–29. doi: 10.1074/jbc.RA118.004255 (PMC6166728; doi:10.1074/jbc.RA118.004255)
Supplement: Supporting Information [file supp_293_39_15316__index.html]

Structure–function analyses of the bacterial zinc metalloprotease effector protein GtgA uncover key residues required for deactivating NF-κB — GtgA mimics DNA to cleave a subset of NF-κB proteins — Supporting Information 

# Structure–function analyses of the bacterial zinc metalloprotease effector protein GtgA uncover key residues required for deactivating NF-κB

## Supporting Information

- Structure of GtgA in complex with p65 explains the substrate specificity of Salmonella zinc metalloprotease effectors - Supporting information Figures S1-8
- PDB summary validation report - PDB report for GtgA
- PDB summary validation report - PDB report for GtgA in complex with p65
