# Supplementary material for: Structure–function analyses of the bacterial zinc metalloprotease effector protein GtgA uncover key residues required for deactivating NF-κB
Source: J Biol Chem. 2018 Jul 26;293(39):15316–29. doi: 10.1074/jbc.RA118.004255 (PMC6166728; doi:10.1074/jbc.RA118.004255)
Supplement: Supporting Information [file supp_RA118.004255_138541_2_supp_173084_pcbpr1.pdf]

Supporting Information

Structure-function analyses of the bacterial zinc metalloprotease effector protein GtgA uncovers key residues required for deactivating NF-κB

Elliott Jennings[1], Diego Esposito[2], Katrin Rittinger[2], Teresa L. M. Thurston[1*]

From the [1]Section of Microbiology, MRC Centre for Molecular Bacteriology and Infection, Imperial College London, London, SW7 2AZ, United Kingdom; [2]Molecular Structure of Cell Signalling Laboratory, The Francis Crick Institute, 1 Midland Road, London, NW1 1AT, United Kingdom

Running title: *GtgA mimics DNA to cleave a subset of NF-κB proteins*

* To whom correspondence should be addressed: Teresa L. M. Thurston: Section of Microbiology, Medical Research Council Centre for Molecular Bacteriology and Infection, Imperial College London, London, United Kingdom; t.thurston@imperial.ac.uk; Tel. (44) 2075943074

Contents:

Figure S1. Sequence alignment of GtgA, GogA, and PipA.
Figure S2. Structural characteristics of Zincin superfamily members.
Figure S3. Relative abundance of p65, p50, RelB-FLAG, FLAG-cREL and FLAG-p100 in 293ET cells expressing the indicated GFP-tagged effector protein.
Figure S4. Cleavage of His_6-SUMO-$p65^{20-188}$ and His_6-SUMO-$p65^{20-291}$ by GtgA
Figure S5. The 19 N-terminal residues of GtgA are not required for GtgA catalytic activity.
Figure S6. Size exclusion chromatography of $GtgA^{20-288\ E183Q}$.
Figure S7. Superimposition of GtgA apo structure with GtgA-p65 complex reveals steric clashes between GtgA residue D139 and p65 residues K37 and C38.
Figure S8. GogA and PipA structural homology models.

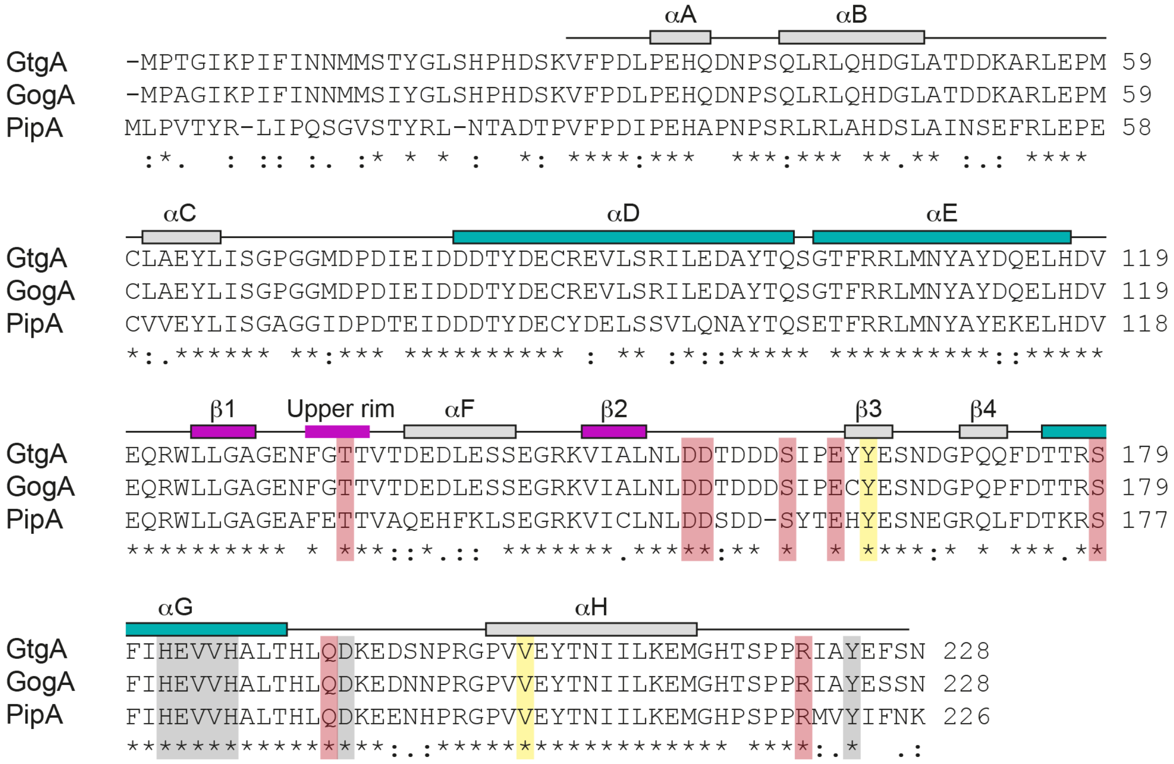

Figure S1. Sequence alignment of GtgA, GogA, and PipA.
Secondary structure of GtgA and sequence alignment of GtgA, GogA, and PipA from *Salmonella* Typhimurium strain ATCC 14028s. The secondary structure diagram is coloured as in Figure 3C; α helices in the Zincin-like catalytic core are coloured teal, whereas strands β1, β2, and the active site upper rim residues are coloured purple. The HEXXH motif, zinc-coordinating aspartate and the conserved tyrosine are highlighted in grey. GtgA residues which form hydrogen bonds with p65 residues in the complex structure are highlighted in red. Lastly, Y165 and V204, which sandwich GtgA residue R221 are highlighted in yellow.

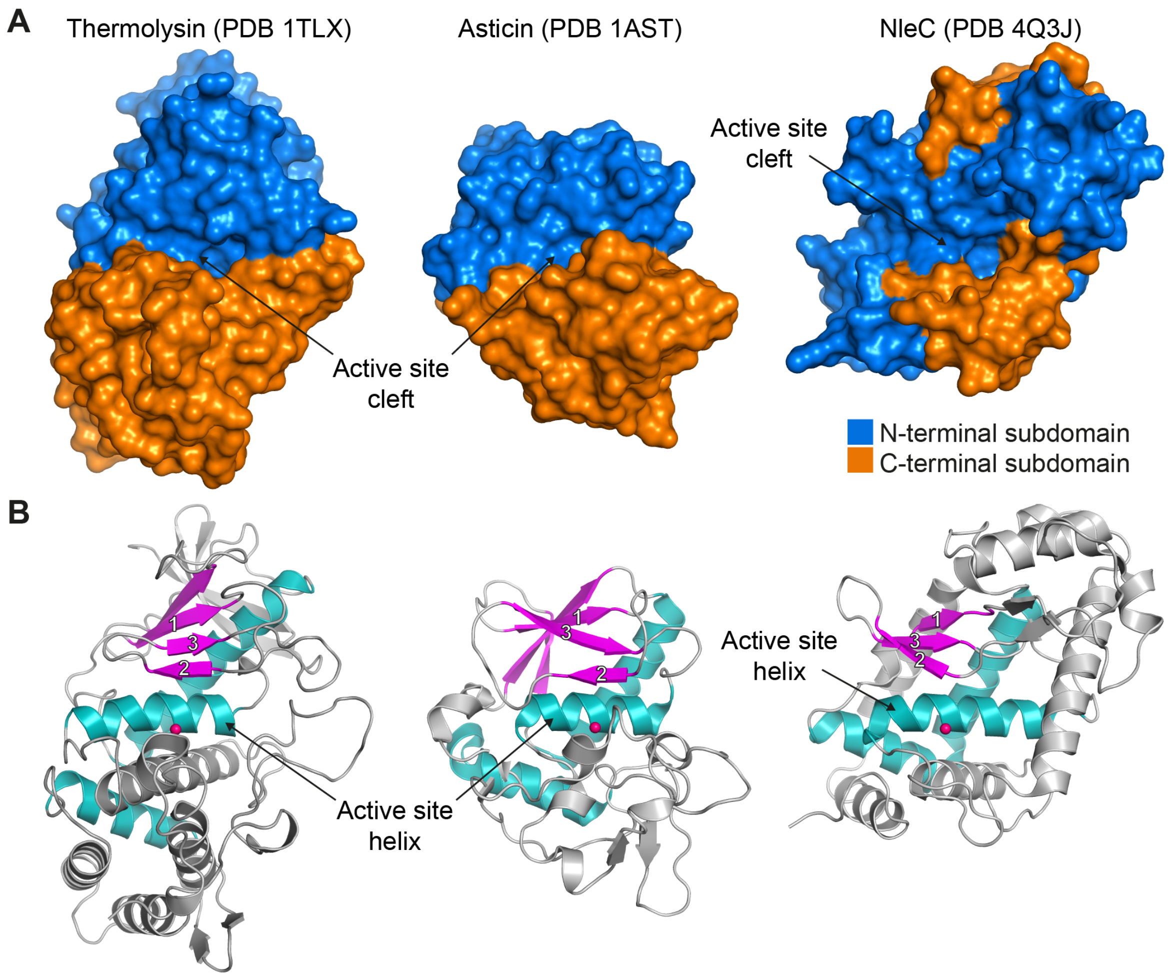

Figure S2. Structural characteristics of Zincin superfamily members.
(A) Surface and (B) cartoon representation of Thermolysin (PDB 1TLX) (1), Asticin (PDB 1AST) (2) and NleC (PDB 4Q3J) (3). In panel B, the β-strands in the N-terminal subdomain are coloured purple and conserved α-helices are coloured teal. The 3 C-terminal strands that form the ψ-loop motif are numbered 1 to 3. The catalytic zinc ions are shown as pink spheres.

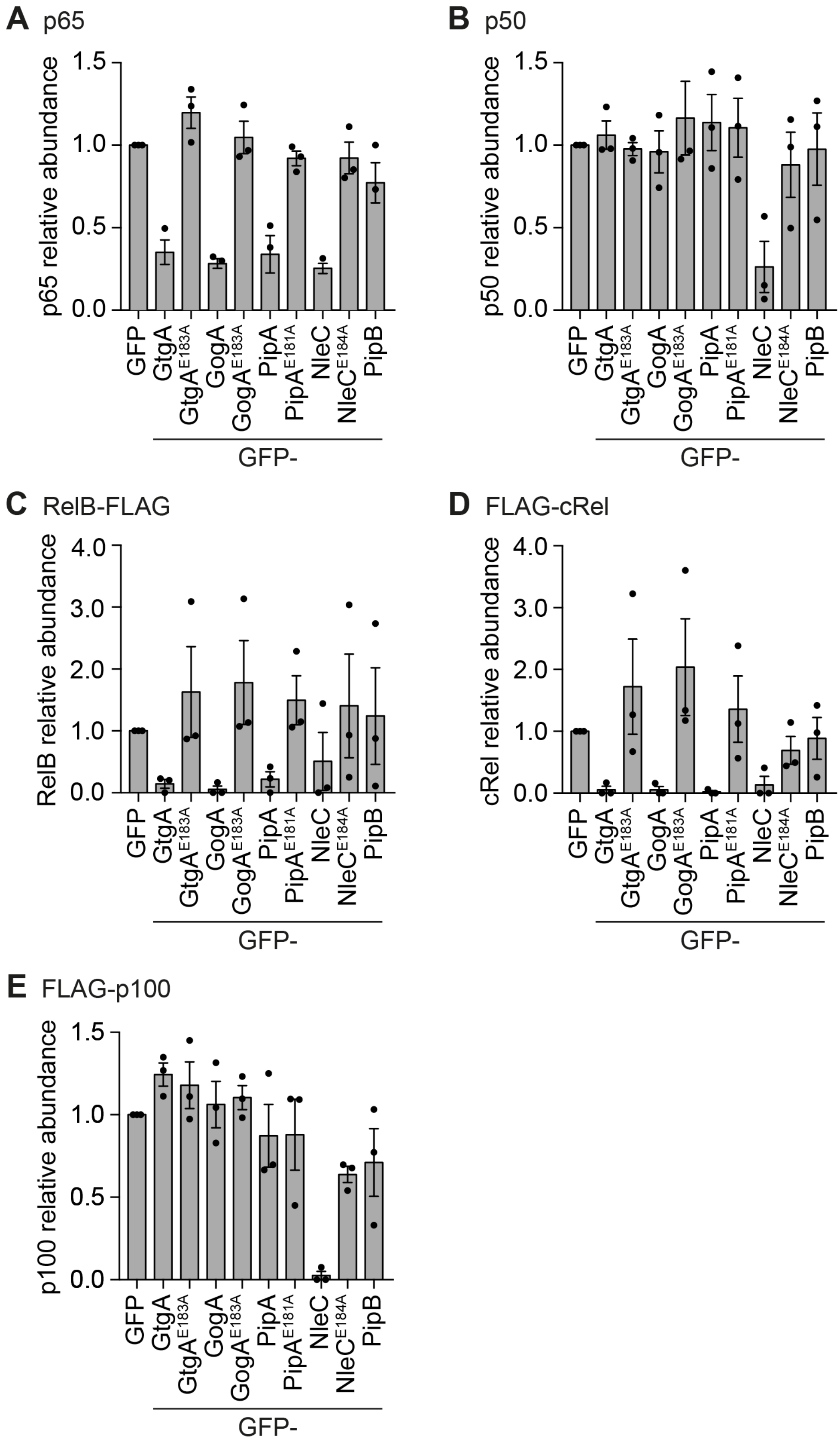

Figure S3. Relative abundance of p65, p50, RelB-FLAG, FLAG-cRel and FLAG-p100 in 293ET cells expressing the indicated GFP-tagged effector protein.
Immunoblots shown in Figure 1A were analysed by densitometry analysis. Data represents the mean ± S.E.M. of 3 independent experiments, for which individual data points are indicated.

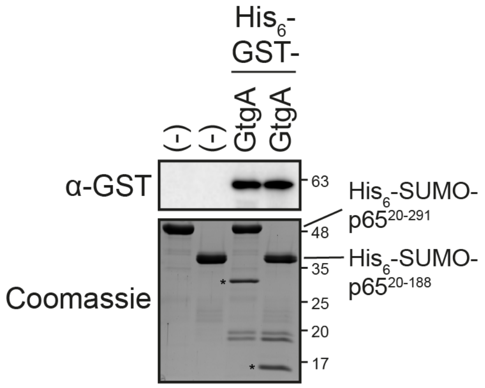

Figure S4. Cleavage of His$_6$-SUMO-p65$^{20\text{-}188}$ and His$_6$-SUMO-p65$^{20\text{-}291}$ by GtgA.
5 μM His$_6$-SUMO-p65$^{20\text{-}188}$ or 5 μM His$_6$-SUMO-p65$^{20\text{-}291}$ was incubated with 0.1 μM of His$_6$-GST-GtgA for 5 hours at 37 °C. The reaction was then quenched by the addition of 2x Laemmli buffer and proteins separated and visualized by SDS-PAGE followed by Coomassie Blue staining. Immunoblot analysis using an anti-GST antibody was done to confirm equal amounts of GST-tagged effector protein. The Coomassie Blue-stained polyacrylamide gel and immunoblot are representative of 3 independent experiments. The * denotes the C-terminal cleavage fragment.

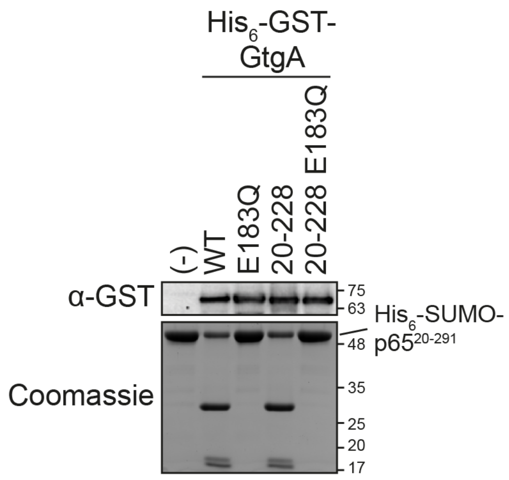

Figure S5. The 19 N-terminal residues of GtgA are not required for GtgA catalytic activity. 5 μM His_6-SUMO-$p65^{20\text{-}291}$ was incubated with 0.1 μM of the indicated His_6-GST-GtgA variant for 5 hours at 37 °C. The reaction was then quenched by the addition of 2x Laemmli buffer and proteins separated and visualized by SDS-PAGE followed by Coomassie blue staining. Immunoblot analysis using an anti-GST antibody was done to confirm equal amounts of each GST-tagged effector protein. The Coomassie Blue-stained polyacrylamide gel and immunoblot are representative of 3 independent experiments.

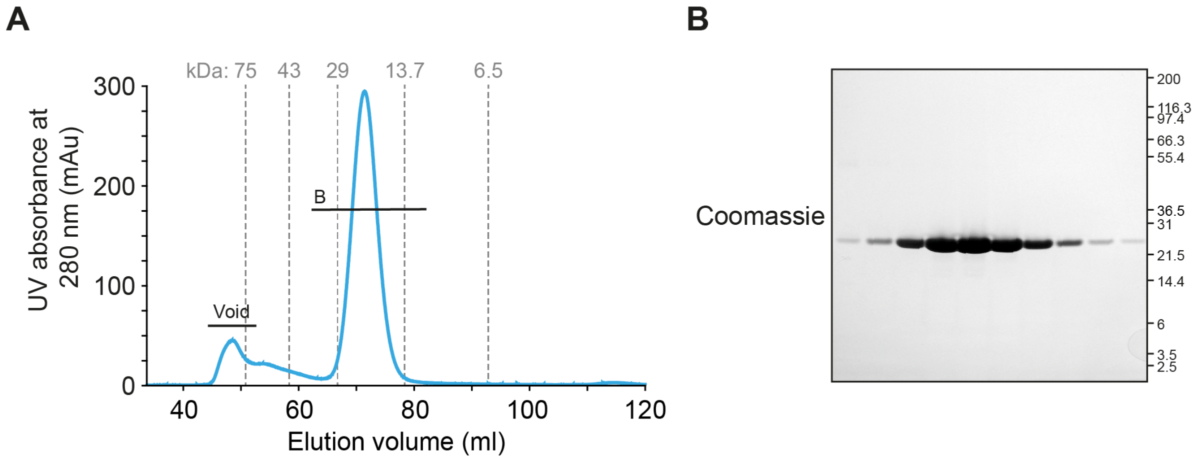

Figure S6. Size exclusion chromatography of GtgA$^{20\text{-}288\ E183Q}$.
(A) Elution profile of GtgA$^{20\text{-}228\ E183Q}$ separated by size exclusion chromatography using a Hi Load 16/60 Superdex 75 size exclusion column. The grey dashed lines depict the calibration curve for standard proteins (GE Healthcare Life Sciences). (B) Peak elution fractions were analysed by SDS-PAGE and Coomassie blue staining.

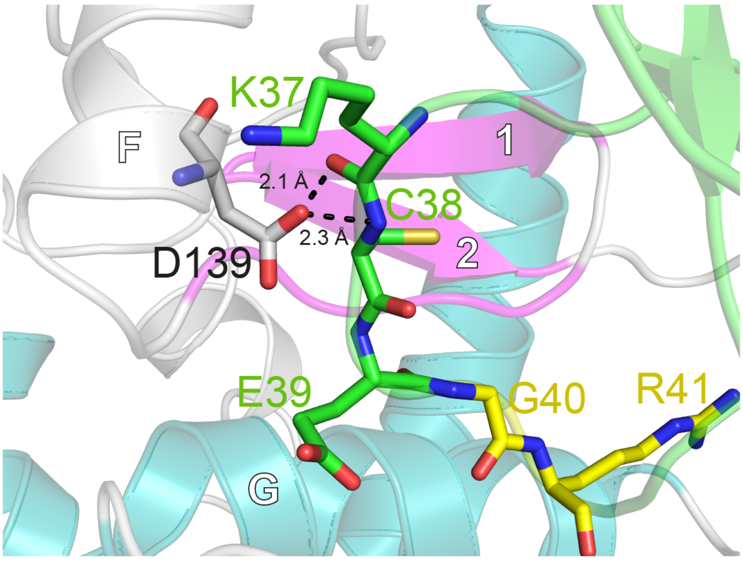

Figure S7. Superimposition of GtgA apo with GtgA-p65 complex reveals steric clashes between GtgA residue D139 and p65 residues K37 and C38.

The GtgA apo structure was aligned with the structure of GtgA in complex with the NTD of p65. Apo GtgA is coloured as in Figure 3C; α-helices are coloured teal, whereas the β1β2 β-sheet and the active site upper rim residues are coloured purple. p65 is coloured green with the exception of the G40/R41 cleavage site which is coloured yellow.

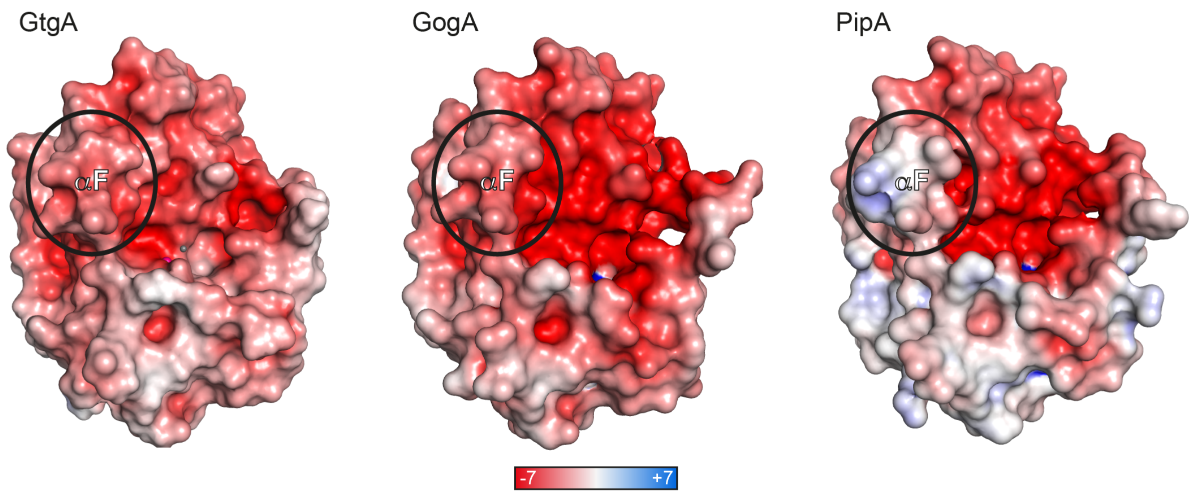

Figure S8. GogA and PipA structural homology models.
Solvent accessible surface representation coloured according to the electrostatic surface potential (positive blue, negative red) of the structure of GogA (97% sequence identity with GtgA) and PipA (67%), as calculated using Adaptive Poisson-Boltzmann Solver (APBS) in PyMOL. The differential electrostatic surface potential of helix αF between GtgA/GogA and PipA is highlighted.

References

1. English, A. C., Done, S. H., Caves, L. S., Groom, C. R., and Hubbard, R. E. (1999) Locating interaction sites on proteins: the crystal structure of thermolysin soaked in 2% to 100% isopropanol. *Proteins* **37**, 628-640
2. Bode, W., Gomis-Ruth, F. X., Huber, R., Zwilling, R., and Stocker, W. (1992) Structure of astacin and implications for activation of astacins and zinc-ligation of collagenases. *Nature* **358**, 164-167
3. Turco, M. M., and Sousa, M. C. (2014) The Structure and Specificity of the Type III Secretion System Effector NleC Suggest a DNA Mimicry Mechanism of Substrate Recognition. *Biochemistry* **53**, 5131-5139
